# Smooth Pursuit Eye-Movement Abnormalities Associated With Cervical Spine Whiplash: A Scientific Review and Case Report

**DOI:** 10.7759/cureus.9872

**Published:** 2020-08-19

**Authors:** Marie Boo, Gordon Matheson, Angela Lumba-Brown

**Affiliations:** 1 Sports Medicine, Stanford University, Stanford, USA; 2 Emergency Medicine, Stanford University, Stanford, USA

**Keywords:** smooth pursuit, eye-tracking, whiplash, cervical, concussion

## Abstract

Whiplash injuries may disrupt normal cervical afferent and efferent projections. Oculomotor abnormalities have been reported in chronic whiplash cases, but there is limited knowledge of their presence in acute whiplash and how acute assessment may target early intervention. We present a literature review and case study of a 22-year-old female presenting with an acute concussion and whiplash secondary to a high-speed motor vehicle collision. Smooth pursuit eye-movement abnormalities were observed in relative cervical rotation in the setting of clinical examination of cervicogenic dysfunction. Treatment was focused on cervical manual therapy. While concussive symptoms resolved after seven days, eye-tracking showed a mild improvement and continued to exist in relationship with cervicogenic dysfunction. After completing physical therapy twice weekly for two weeks and in-home exercises, clinical signs and symptoms of whiplash-associated cervicogenic dysfunction and abnormal smooth pursuit eye-movement resolved across all cervical positions. This case highlights the need for ocular-motor impairment assessment following acute whiplash, specifically during cervical rotation. Early intervention should focus on cervical manual therapy and may be important in supporting altered cervical afferents causing oculomotor dysfunctions following acute whiplash.

## Introduction

Traumatic cervical strain, including whiplash, may result in altered sensorimotor control secondary to mechanisms involving pathophysiologic afferent input related to pain and inflammation and the extensive integration of cervical afferents and efferents within the central nervous system [1]. Whiplash-associated disorders (WADs) result in musculoskeletal dysfunction and symptoms including: static and functional neck pain and stiffness, shoulder and extremity pain, paresthesia, fatigue, dizziness, tinnitus, sleep disruption, neurocognitive disruption, irritability, mood disturbance, and visual disturbance. WAD often occurs in the setting of acceleration-deceleration impacts following motor vehicle accidents, but can occur via other mechanisms as well [2]. More than 70% of patients with WAD have complaints of dizziness and 50% report visual and balance disturbances, even in the absence of traumatic brain injury [3-5]. Notably, symptom severity has been reported to be higher in patients with trauma-induced neck pain as compared to those with non-traumatic neck pain [6].

An understanding of the neuro-pathophysiologic manifestation of the clinical signs and symptoms of WAD is key to successfully rule out pertinent differential diagnoses and establish an appropriate management plan. Subjective and objective measures, such as the smooth pursuit neck torsion (SPNT) test, are useful to diagnose WADs. The SPNT test quantifies objective eye-movements and can be used to aid the diagnosis of WAD with dizziness [7,8]. This test assesses visual performance during eye-tracking, specifically the variability of gaze positional error relative to a predictably moving target and can be quantified with several parameters [9]. Poor tracking performance is demonstrated by large and variable amplitudes of forward saccades, often seen after a concussion with subsequent attention impairments. Whereas healthy subjects should have no consistent positional errors [9,10]. The objective SPNT test differentiates symptoms of traumatic musculoskeletal WAD from concussion-related vestibular etiologies.

While oculomotor dysfunction has been associated with chronic neck pain, including chronic WAD, limited data exists describing objective oculomotor impairment following WAD in the acute setting - representing an important early therapeutic target [4-8,11,12]. The current case report details WAD and concussion in a collegiate athlete following high-speed motor vehicle accidents and aims to review clinical evaluation, the use of smooth pursuit eye-tracking in diagnosis and treatment, and return-to-sport considerations.

## Case presentation

Informed consent was obtained from the patient for the use of her information, clinical progression, and eye-tracking results. Institutional review board approval is waived at the study’s institution for case reports.

A 22-year-old female varsity soccer player sustained head and neck injury following a high-speed (75 miles per hour) motor vehicle collision. The patient was transported by emergency medical services to the ED with a Glasgow Coma Scale score of 15 and no neurologic deficits. The patient denied the initial loss of consciousness, nausea, vomiting, seizures, or amnesia. Cervical spine plain films were obtained and were negative. Neuroimaging was not obtained. She was discharged from the Emergency Department the same day for next day follow-up with her team physicians. The following day, the patient was evaluated by her team’s sports medicine physician, diagnosed with a concussion and cervical strain secondary to WAD, and referred to physical therapy for vestibular and cervical evaluation. Her management plan, to begin that day, included supervised, sustained cardiovascular exercises for 20-30 minutes with heart rate greater than 140 beats per minute. The patient’s past medical history was significant for concussion two years prior which resolved in one week. She had no prior neck injuries.

Follow-up

Two days post-injury, at her physical therapy appointment, the patient reported headache, cranial pressure, fatigue, neck pain, difficulty with reading, and poor concentration. She denied sleep disturbance, tinnitus, photophobia, diplopia, dysphagia, facial or upper extremity paresthesias, or weakness. Cervical examination revealed negative vertebrobasilar insufficiency bilaterally as well as negative upper cervical instability testing. Cervical active range of motion (ROM) was moderately limited (extension > side bending bilaterally > rotation bilaterally) with mild pain; rotation measured 65 degrees bilaterally. No restrictions or hypomobility in cervical spine joint mobility. She had positive cervicogenic dizziness (seated body-on-head rotation) and significant tenderness to palpation at the suboccipital, cervical paraspinal, anterior, middle, and posterior scalene, and sternocleidomastoid muscles bilaterally. Passive range of motion and joint play assessment was within normal limits, but guarded.

**Figure 1:**
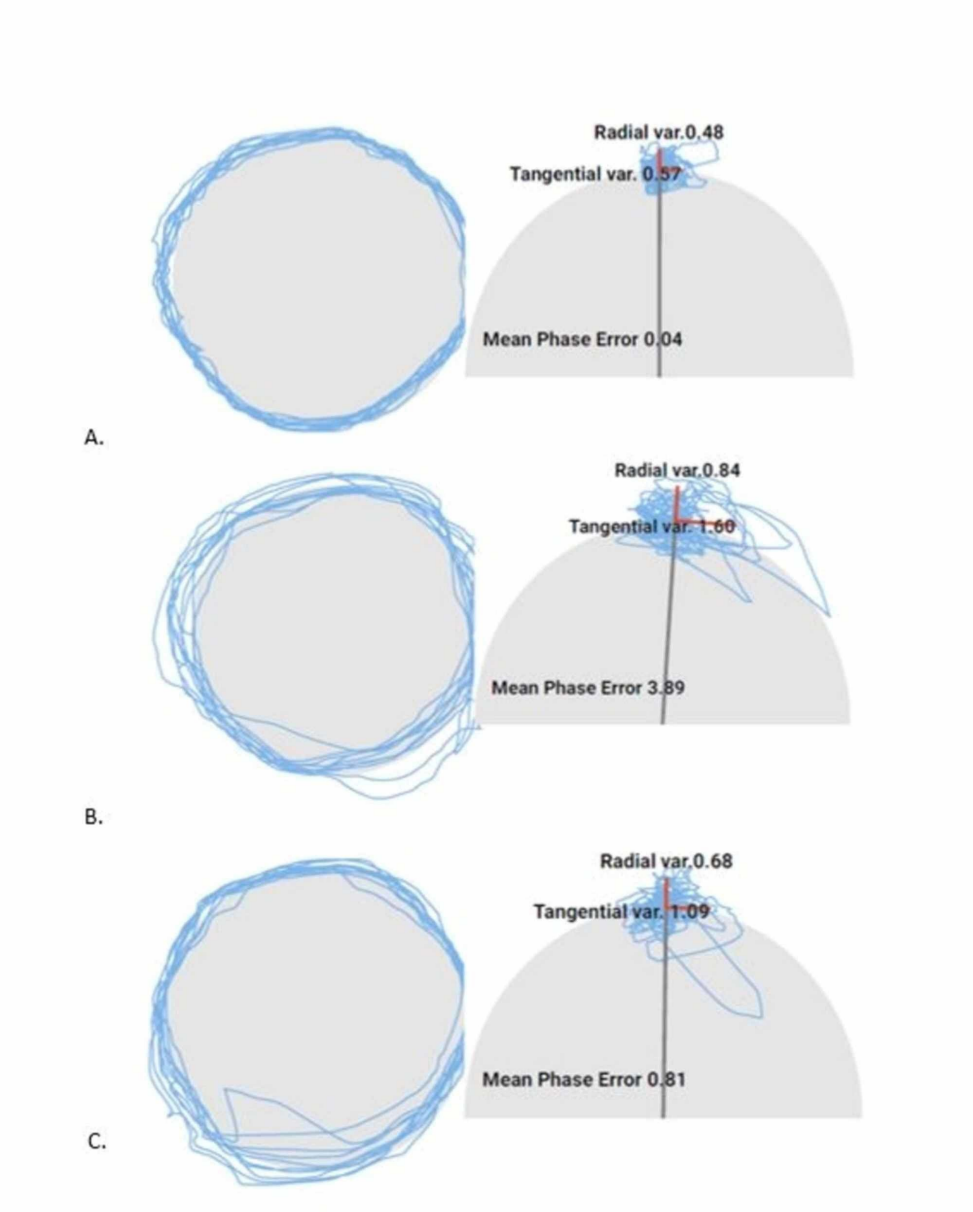
Eye-tracking three days after accident. (A) neutral cervical spine; (B) smooth pursuit neck torsion test, relative right neck rotation; and (C) smooth pursuit neck torsion test, relative left neck rotation.

Vestibular-ocular assessment revealed dysfunctions with horizontal and vertical vestibular ocular reflex (VOR), visual motion sensitivity, dynamic visual acuity (horizontal), and balance (Balance Error Scoring System test = 19/60). Oculomotor examination (including saccades and near-point convergence), positional vertigo tests, and head thrust test were all normal without nystagmus, saccadic, or dysmetric eye movements. Eye-tracking in neutral cervical position measured a standard deviation of tangential error (SDTE) = 0.57, standard deviation of radial error (SDRE) = 0.48, and mean phase error (MPE) = 0.04 as can be seen in Figure 1A and Table 1, without blurry vision. Eye-tracking was then repeated in conjunction with the SPNT test with 45 degrees of relative right and left cervical rotation measuring SDTE = 1.60, SDRE = 0.84, MPE = 3.89 and SDTE = 1.09, SDRE = 0.68, and MPE = 0.81 respectively (Figure 1B, 1C) with concomitant report of blurry vision with body rotation bilaterally [7].

**Table 1:** Parameters of eye-tracking with neutral cervical position and SPNT testing throughout treatment SPNT, smooth pursuit neck torsion; SDTE, standard deviation of tangential error; SDRE, standard deviation of radial error; MPE, mean phase error

		3 days	7 days	14 days (Discharge)
Neutral cervical spine	SDTE	0.57		0.53
	SDRE	0.48		0.51
	MPE	0.04		-2.44
Relative right cervical rotation	SDTE	1.60	0.75	0.64
	SDRE	0.84	0.44	0.59
	MPE	3.89	-2.53	-4.45
Relative left cervical rotation	SDTE	1.09	0.83	0.58
	SDRE	0.68	0.70	0.33
	MPE	0.81	0.17	-2.99

Intervention

Treatment, beginning on day two post-injury, focused on extensive manual therapy of the cervical musculature to decrease tone, decrease pain, and improve active range of motion. A variety of manual therapy techniques were employed including soft-tissue mobilization, trigger point release, manual cervical traction, and mobilizations at the cervico-thoracic junction, thoracic spine, and first rib bilaterally. After manual therapy, the patient then performed deep cervical flexor activation exercises as well as light cervical strengthening to ensure cervical stabilization throughout body positions requiring increasing postural demands. Due to time constraints in clinic, the patient performed exercises for concussive vestibular-ocular dysfunctions at home daily. She also continued non-impact cardiovascular training and progressed to supervised light weight-lifting (symptom-free), consistent with the National Collegiate Athletic Association (NCAA) stepwise progression for return to activity after concussion.

After one day of in-clinic therapy, four days of a home exercise program, and a total of seven days post-injury, the patient’s postconcussive symptoms and all vestibular-ocular dysfunctions resolved, including balance dysfunctions. She also had no symptoms upon exertion with stationary biking or weightlifting. The patient was cleared of concussion by the athletic team’s neurologist and sports medicine physician and was allowed to continue progressing through a stepwise return to activity protocol supervised by the team’s athletic trainer. At this time, though the patient reported no neck pain at rest, she endorsed persisting mild-moderate cervical hypertonicity with concomitant reduced cervical active range of motion. SPNT eye-tracking was repeated in the setting of right and left cervical rotation with report of mild blurry vision with body rotation bilaterally (Right: SDTE = 0.75, SDRE = 0.44, MPE = -2.53 and Left: SDTE = 0.83, SDRE = 0.70, MPE = 0.17) (Table 1). The patient continued physical therapy twice weekly, focusing primarily on the cervical manual therapy outlined above.

Outcome

**Figure 2:**
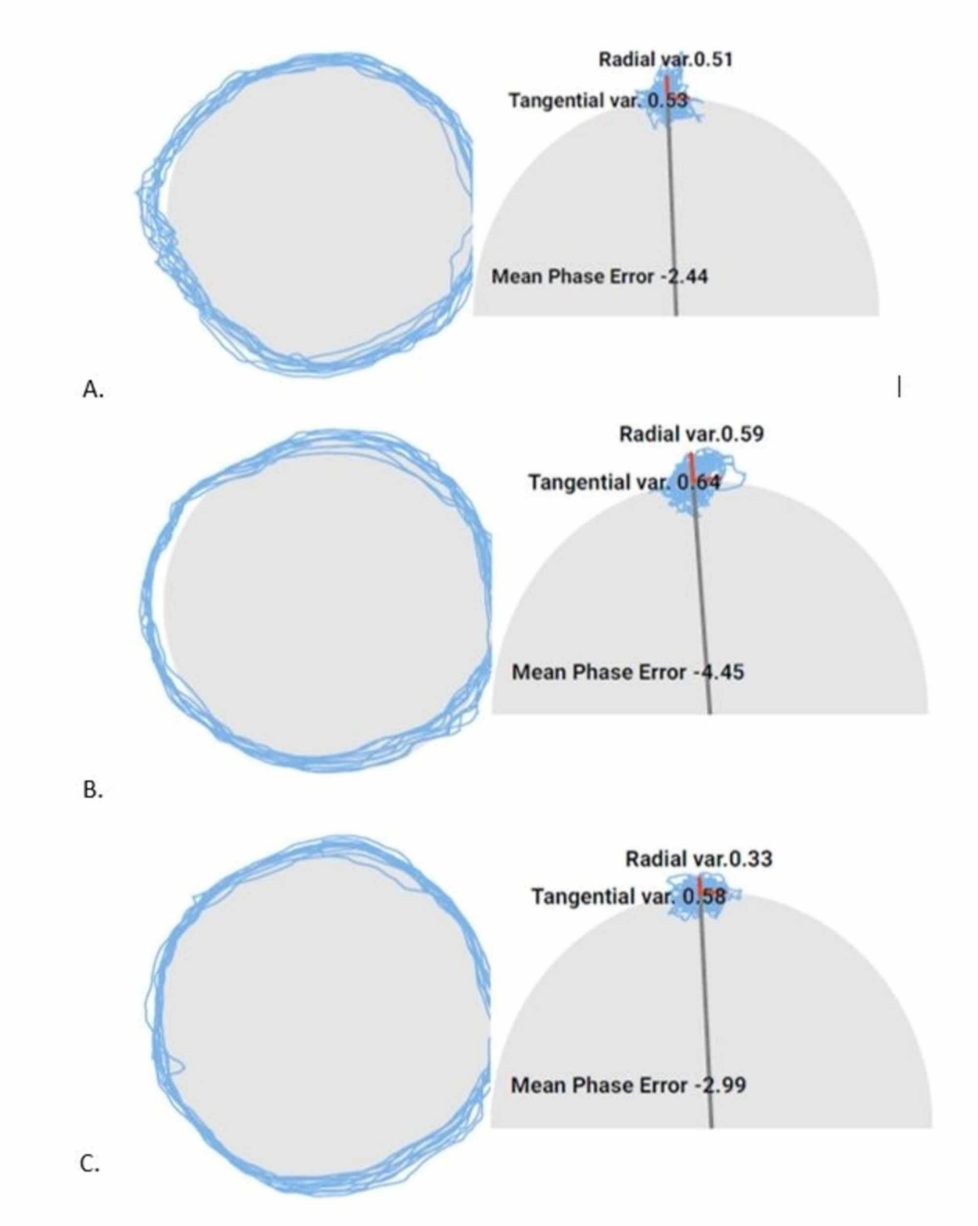
Eye-tracking at discharge. (A) neutral cervical spine; (B) smooth pursuit neck torsion test, relative right neck rotation; and (C) smooth pursuit neck torsion test, relative left neck rotation.

Two weeks post-injury, the patient was discharged from physical therapy with no neck pain and minimal-no restrictions in active range of motion. Repeat eye-tracking was not associated with blurry vision in neutral cervical spine and was reported as SDTE = 0.53, SDRE = 0.51, and MPE = -2.44 and SPNT eye-tracking was reported as SDTE = 0.64, SDRE = 0.59, MPE = -4.45 and SDTE = 0.58, SDRE = 0.33, and MPE = -2.99 with relative right and left cervical rotation respectively (Figure 2). The variability between neutral and rotated cervical spine position is within normal testing variability.

## Discussion

This case report illustrates the use of eye-tracking in conjunction with physical examination for the diagnosis of cervical dysfunction after acute whiplash, and highlights the need for assessment in both concussion and WAD. This case demonstrates a close association between improvements in smooth pursuit oculomotor measurements with subjective and objective clinical progression including neck pain, hypertonicity, and active range of motion. Early intervention of manual therapy allowed the patient to make a full recovery and return to sport in two weeks, thus significantly decreasing incidence of chronic neck pain and/or chronic WAD and the sequelae of further symptoms commonly seen after a high-speed motor vehicle accident. This is consistent with previous work by Rosenfeld et al., which demonstrated early (within 14 days) active intervention, significantly improved pain and overall outcomes compared to a control group at six months and three years after WAD [13]. Eye-tracking technology can be especially useful as a quantitative measure of changes in patients’ visual-tracking performance which may indicate a change in the person’s neurological state, particularly as it relates to attention dysfunction which has been shown to be impaired after a concussion [10]. The patient’s concomitant concussion with vestibular symptoms not explained by WAD and cervical contributions, most notably visual motion sensitivity, was also treated but is beyond the scope of this case report.

The findings of the current case report are in conjunction with previously published articles demonstrating smooth pursuit dysfunctions in patients with whiplash and neck pain [4-6,11,12]. However, to the authors’ knowledge, this is the first case report detailing the correlation acutely after injury. This is important as previous studies have hypothesized this dysfunction may be secondary to prolonged changes in range of motion causing increased cervical muscular activity, altering proprioception, and ultimately resulting in oculomotor dysfunctions [2,3]. This case report, therefore, shows that these changes do not need to be chronic in nature to have oculomotor abnormalities. Furthermore, this supports other hypotheses that changes in smooth pursuit during neck torsion may result from a combination of neurophysiological contributions from nociceptive, proprioceptive, and mechanoreceptive afferents [5-7,12]. Additionally, reflexes originating in the cervical spine cannot be dismissed as other contributors. For instance, with the cervical spine in rotation there is altered tension on the cervical muscles not experienced in a neutral position; this may also affect cervico-collic reflex (CCR) and cervico-ocular reflex (COR) afferents which could contribute to abnormal afferents and ultimately oculomotor changes [7,11].

As evidenced by this case, manual therapy may mitigate the above neurophysiological contributions by decreasing pain, decreasing hypertonicity, and improving cervical ROM thereby ameliorating the abnormal afferents. The current case report focused only on manual therapy to better elucidate the effect of one treatment technique on acute whiplash. However, focus on proprioceptive training in isolation or in conjunction with other manual therapy techniques, including the addition of upper cervical spine joint mobilizations or manipulations, may lead to different results in timeline, pain, ROM, and/or eye-tracking. In addition, it is important to note that the patient not only had changes in smooth pursuit tangential variable during the SPNT test, but she also had a positive cervicogenic dizziness test as well as visual disturbances with cervical rotation likely due to abnormal cervical afferent input and ultimately altered sensorimotor control [1]. These may be important designations for the diagnosis of cervical contribution in the absence of eye-tracking technology.

## Conclusions

The current case report shows the clinical utility of eye-tracking technology to assist with diagnosing the contribution of cervical dysfunctions in patients with acute WAD, in the setting of concussion - as in this case, or in isolation. Furthermore, in combination with the SPNT test, eye-tracking is useful in monitoring the correlation of eye-tracking with the patient’s clinical progression. Early intervention with focus on cervical manual therapy may be important in normalizing the altered cervical afferents that cause oculomotor control dysfunctions seen after acute whiplash.
